# Effect of Nonalcoholic Fatty Liver Disease (NAFLD) on COVID-19: A Single-Center Study of 3983 Patients With Review of Literature

**DOI:** 10.7759/cureus.26683

**Published:** 2022-07-09

**Authors:** Preetam Nath, Raj Kumar, Bipadabhanjan Mallick, Swati Das, Anil Anand, Sarat C Panigrahi, Ajay Duseja, Subrat K Acharya, Yogesh K Chawla, Dibya L Praharaj

**Affiliations:** 1 Gastroenterology and Hepatology, Kalinga Institute of Medical Sciences, Bhubaneswar, IND; 2 Radiodiagnosis, Kalinga Institute of Medical Sciences, Bhubaneswar, IND; 3 Hepatology, Post Graduate Institute of Medical Education and Research, Chandigarh, IND

**Keywords:** coronavirus disease 2019, nonalcoholic fatty liver disease (nafld), duration of hospital stay, mortality, outcome, fatty liver, covid-19

## Abstract

Background

The presence of metabolic syndrome (MS) is associated with increased disease severity in patients with coronavirus disease 2019 (COVID-19). Non-alcoholic fatty liver disease (NAFLD) associated with or without MS may be related to increased morbidity and mortality in COVID-19, but large Indian studies are lacking. The present study was carried out to assess the impact of NAFLD on the clinical outcomes in patients with COVID-19 infection.

Methods

All patients with COVID-19 hospitalized at a tertiary care hospital in eastern India from April 4 to December 31, 2020, were included in the study. Patients who underwent non-contrast CT (NCCT) chest were evaluated for the presence of hepatic steatosis based on a validated criterion liver attenuation (HU) value lower than the spleen, absolute liver attenuation lower than 40 HU, and liver to spleen attenuation ratio less than 1. Patients were divided into two groups, those with or without fatty liver. Baseline characteristics including age, sex, liver function tests, and outcomes including duration of hospital stay and mortality were compared.

Results

A total of 6003 COVID-19-positive patients were admitted during the study period. Of these patients, 214 children (<18 years) with COVID-19 infection were excluded. One hundred and eight patients with a history of significant ethanol abuse were excluded from the analysis. NCCT scan was not done in 1698 patients. Finally, 3983 patients were included in the study. They were divided into two groups depending on the presence or absence of NAFLD. Of the 3983 patients, 814 (20.4%) had NAFLD. Overall in-hospital mortality among the study group was 6.4%. The mortality rate among patients with NAFLD was 6.7% while that in patients without fatty liver was 6% (P=0.381). Similarly, the mean duration of hospital stay was also comparable between both the groups (10.63±7.2days vs 10.65±6.6 days;P=0.66). Prevalence of NAFLD was similar in survivors and non-survivors; 759 of 2981 patients (25.4%) and 55 of 188 patients 29.2% (P=0.381), respectively. On univariate analysis, male sex, older age, elevated alanine aminotransferase (ALT), aspartate aminotransferase (AST), and gamma-glutamyl transpeptidase (GGT) along with low serum albumin and low absolute eosinophil counts (AEC) were associated with higher mortality. However, on multivariate analysis, only older age, male sex, and low albumin levels were associated with higher mortality. Surprisingly, a sub-group analysis showed that females without NAFLD were at a higher risk of mortality than those with fatty liver (4.9% vs 12.3%; P=0.006). Similarly, patients with lower AST levels had higher mortality compared to patients with significantly elevated AST levels (more than two times the upper limit of normal (ULN)), irrespective of the presence of fatty liver.

Conclusions

The prevalence of fatty liver in severe acute respiratory syndrome coronavirus 2 (SARS CoV-2) infected patients is similar to the general population in India, the presence of which is not a predictor of severe disease. However, mortality is higher in males and elderly patients.

## Introduction

This preliminary results of the study was previously presented as poster at the 28th INASL Annual Scientific Meeting on August 06, 2021.

Coronavirus disease 2019 (COVID-19) infection was first reported in Wuhan city of China in December 2019 and later affected individuals all over the world [1]. As of July 7, 2022, 55,02,18,992 individuals had been infected with the deadly virus with about 63,43,783 individuals succumbing to the illness [2]. In 2020, WHO declared COVID-19 infection as a global health emergency and urged nations to work together to tackle the fatal illness [3]. Though about 80% of the patients have mild illness and recover with symptomatic treatment, few unfortunate individuals develop severe disease and succumb to the illness even with optimal care [4]. Reports have suggested that the disease tends to be severe in elderly individuals and in patients with metabolic syndrome (MS) [5]. During the ongoing pandemic of COVID-19, the prevalence of another chronic liver disease is being increasingly reported worldwide. Non-Alcoholic Fatty Liver Disease (NAFLD), the hepatic manifestation of MS, currently constitutes the most common chronic liver disease worldwide [6]. In India, 9-32% of the population have NAFLD [7]. A few small studies have shown that the presence of NAFLD per se is an independent risk factor for acquiring COVID-19 infection. A few, but not all, studies have also reported higher morbidity and mortality [8,9]. In a small case-control study conducted at a tertiary care hospital in India, Madan et al. found no association between the presence of fatty liver and COVID-19-associated clinical outcomes (Morbidity and mortality) [10]. However, large studies showing an association between the presence of fatty liver and the severity of COVID-19 infection are lacking at present. We had a unique opportunity to manage over 6000 COVID-19 infected patients admitted during the first wave of the pandemic in 2020 in our hospital. The aim of our study was to evaluate the relationship between NAFLD and COVID-19 infection using clinical and laboratory data of individuals admitted to a tertiary care hospital in eastern India diagnosed with COVID-19.

## Materials and methods

This was a prospective observational study conducted at the Kalinga Institute of Medical Sciences (KIMS) and Pradyumna Bal Memorial (PBM) Hospital, Bhubaneswar, Odisha, India, from April 4 to December 31, 2020. The study protocol was approved by the Institutional Ethics Committee (IEC), Kalinga Institute of Medical Sciences (KIMS), Kalinga Institute of Industrial Technology (KIIT) deemed to be university, Bhubaneswar, India (KIIT/KIMS/IEC/523/2020). The primary outcome measure of the study was mortality in COVID-19 infected patients with or without fatty liver while the secondary outcome measure was the total duration of hospital stay.

Inclusion and exclusion criteria

All adult patients (age≥ 18 years) who were diagnosed as COVID-19 positive, admitted into our COVID-19 hospital from April 4 to December 31, 2020, and underwent a non-contrast CT (NCCT) scan of the chest were included in the study. All these images were acquired in a craniocaudal direction (from the thoracic inlet to the mid-portion of kidneys) at shallow inspiration. This enabled us to acquire images of both the spleen and liver.

All patients less than 18 years of age, patients with prior history of cirrhosis (compensated or decompensated), and/or significant ethanol abuse were excluded. Patients who didn’t undergo an NCCT scan were also excluded.

Study definitions

The diagnosis of COVID-19 infection was based on either a positive reverse transcriptase-polymerase chain reaction (RT-PCR) or rapid antigen test (RAT). Fatty liver was diagnosed on the basis of the following radiological parameters in the NCCT scan of the chest performed as part of a workup done in sick patients with COVID-19 infection: Liver attenuation value lower than the spleen, absolute liver attenuation of less than 40 HU and liver to spleen attenuation ratio less than 1 [11]. Liver attenuation was calculated by placing the circular region of interest (ROI) of at least 1 cm² area at multiple places in the liver, covering all the hepatic segments. Care was taken to avoid the inclusion of macroscopic vessels and areas close to fissures during attenuation measurements. Splenic attenuation was measured by placing ROI at its upper, mid, and lower poles. Average of a large number of ROIs more than nine were taken to minimize the effect of heterogeneity. The following criteria were used to diagnose NAFLD: the presence of fatty liver as per CT scan and absence of a history of excessive alcohol consumption as per the clinical records. (defined as average alcohol intake ≥ 30 grams/day in men and ≥ 20 grams/day in women) [12]. 

Data collection

The individual medical records of all the patients were analyzed. Information regarding demographic data, hemoglobin, total leukocyte count (TLC), platelet count (PC), coagulation parameters and biochemical parameters (liver functions and renal functions), duration of hospital stay, and final outcome (death or discharge) were populated in the master chart. All the CT scans were analyzed for the presence of fatty liver by an experienced hepato-radiologist. The imaging of the liver and spleen in the NCCT chest from the Picture Archiving and Communication System (PACS) of KIMS and PBM Hospital was used to detect fatty liver in these patients.

Statistical analysis

Categorical data were presented as (n, %); Continuous data were written in form of mean ± SD or median± interquartile range (IQR) as appropriate. The Chi-square test was performed for categorical variables while the student-t-test was performed for continuous variables. For skewed data, a non-parametric Mann-Whitney U- test was used for the analysis of the two groups. Multivariate regression analysis was performed to estimate the odds ratio for variables on the severity of COVID-19. All statistical tests were two-sided and were performed at a significance level of α=0.05. Statistical analysis was performed using IBM SPSS Statistics for Windows, Version 22.0 (Released 2013; IBM Corp., Armonk, New York, United States).

## Results

**Figure 1:**
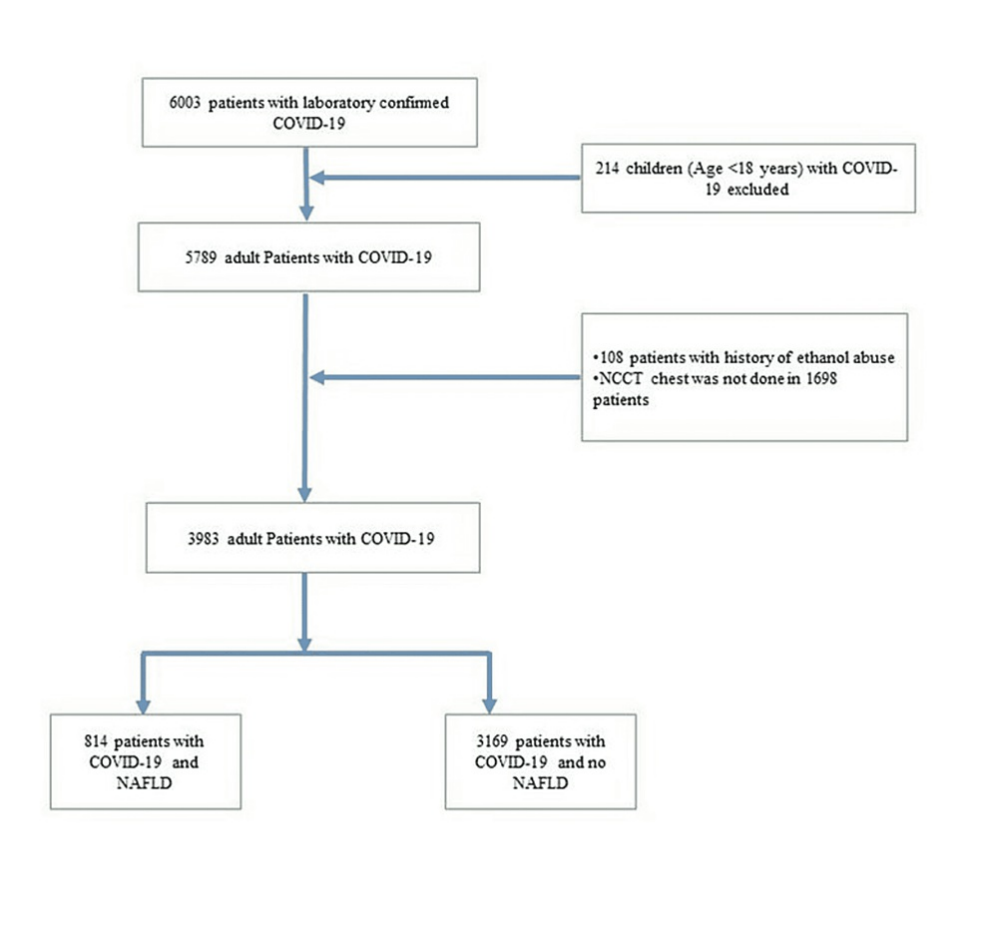
Flow chart of the study population NCCT: non-contrast CT; NAFLD: non-alcoholic fatty liver disease; COVID-19: coronavirus disease 2019

A total of 6003 patients were admitted to the COVID-19 hospital of KIMS, PBM hospital, between April 4 and December 31, 2020. Of these, 218 patients belonging to the pediatric age group (age <18 years) were excluded; 108 patients had a history of excessive alcohol intake while an NCCT scan of the chest was not done in 1698 patients. Finally, a total of 3983 patients with COVID-19 were included in the study (Figure 1).

Based on NCCT chest studies, 814 patients (20.4%) were diagnosed to have NAFLD while 3169 patients didn’t have NAFLD. Thus the prevalence of NAFLD in the COVID-19 population was at par with the Indian studies reported previously [7]. In both NAFLD and non-NAFLD groups, male preponderance was noted. The mean age of patients in the NAFLD group was significantly higher compared to the non-NAFLD group (47.11 ± 14.34 years vs 45.18 ± 16.06 years; p= 0.002). Most of the admitted patients in both groups were males. The prevalence of diabetes mellitus (DM) was comparable across both groups. Length of hospital stay and mortality were also similar in patients with or without NAFLD.

With regard to the laboratory findings, TLC, absolute neutrophil count (ANC), absolute lymphocyte count (ALC), and absolute eosinophil count (AEC) were comparable in both groups. Inflammatory markers like C-reactive protein (CRP), ferritin, and D-dimer levels were also similar. Liver lab parameters including serum bilirubin, albumin, and Alkaline Phosphatase (ALP) levels were comparable across both groups. In contrast, serum aspartate transaminase (AST), alanine transaminase (ALT), and gamma-glutamyl transpeptidase (GGT) were higher in the NAFLD group compared to the non-NAFLD group. A detailed comparison of these baseline characteristics has been provided in Table 1.

**Table 1:** Baseline characteristics and outcome of patients in both the groups NAFLD: non-alcoholic fatty liver disease; IQR: interquartile range; ANC: absolute neutrophil count; ALC: absolute lymphocyte count; AEC: absolute eosinophil count; AST: aspartate transaminases; ALT: alanine transaminases; ALP: alkaline phosphatase; GGT: gamma-glutamyl transpeptidase; TSB: total serum bilirubin; WBC: white blood corpuscle; CRP: C-reactive protein

Baseline Characteristics and outcomes	NAFLD (n=814)	Non-NAFLD (n=3169)	P-value
Sex (Males), n (%)	673 (82.6%)	2311 (72.9%)	0.000
Age (yrs), (Mean ± SD)	47.1± 14.3	45.18 ± 16.06	0.002
Diabetes Mellitus (Yes), n (%)	72 (43.3%)	149 (43.5%)	0.970
WBC x 10^9^/L, (Mean ± SD)	8.2±4.2	7.9±4.21	0.07
TSB (mg/dL), Median (IQR)	0.53 (0.37-0.73)	0.48 (0.32-0.69)	0.143
AST (U/L), Median (IQR)	47.0 (37-67)	39.0 (30.0-56.0)	<0.001
ALT (U/L), Median (IQR)	46.0 (29-71)	30.0 (30.0-53.0)	<0.001
ALP (U/L), Median (IQR)	81.0 (64-102)	80.0 (64.0-101.0)	0.324
GGT (U/L), Median (IQR)	53.0 (32-98)	30.0 (19.0-57.0)	<0.001
Albumin (g/dL), Median (IQR)	4.0 (3.6-4.4)	4.0 (3.6-4.4)	0.665
CRP (mg/L), Median (IQR)	13.37 (4.24-51.36)	16.99 (5.0-60.5)	0.126
D-Dimer (µg/mL), Median (IQR)	0.69 (0.44-1.19)	0.69 (0.43-1.21)	0.727
ANC x 10^9^/L, Median (IQR)	4.520 (0.20-22.6)	4.5 (0.033-45.09)	0.926
ALC x 10^9^/L, Median (IQR)	1.976 ± 1.427	2.025 ± 1.812	0.364
AEC x 10^9^/L ,Median (IQR)	0.127 (0.006-2.55)	0.134 (0.006-5.712)	0.369
Ferritin (µg/L), Median (IQR)	164 (0.040-1019)	160 (0.020-10000)	0.721
Length of hospital stay (days) (Mean ± SD)	10.63±7.2	10.65±6.6	0.447
Mortality (n,%)	55 (6.7)	188 (5.9)	0.381

With respect to final clinical outcomes, there was no significant difference between NAFLD and non-NAFLD patients. A total of 243 (6.4%) patients died during a hospital stay. The mortality rate was comparable between the two groups (54 (6.7%) patients in the NAFLD group and 188 (6%) in the non-NAFLD group; p=0.381). Similarly, the mean duration of hospital stay was also comparable (10.63±7.2 days in the NAFLD group and 10.65±6.6 days in the non-NAFLD group; p=0.447). Due to logistics reasons, the mean duration of ICU stay between the two groups could not be compared. Based on final outcome, patients were divided into two groups and were compared as per baseline clinical and laboratory parameters (Table 2).

**Table 2:** Baseline clinical and laboratory parameters associated with in-hospital mortality among patients with COVID-19 pneumonia IQR: interquartile range; ANC: absolute neutrophil count; ALC: absolute lymphocyte count; AEC: absolute eosinophil count; AST: aspartate transaminases; ALT: alanine transaminases; ALP: alkaline phosphatase; GGT: gamma-glutamyl transpeptidase; TSB: total serum bilirubin; WBC: white blood corpuscle; CRP: C-reactive protein; COVID-19: coronavirus disease 2019

Baseline parameters	Died (n=243)	Survived (n=3740)	P-value
Sex (Male), n (%)	201 (82.7%)	2783 (74.4%)	0.004
Age (yrs) (Mean ± SD)	59.53±13.8	44.6±15.4	0.000
Diabetes mellitus (Yes), n (%)	22 (47.8%)	306 (43.2%)	0.541
WBC x 10^9^/L(Mean ± SD)	8.6±4.2	8.0±4.21	0.075
TSB, (mg/dL), Median (IQR)	0.31 (0.3-0.72)	0.5 (0.33-0.7)	0.227
AST, (U/L), Median (IQR)	58 (44-82)	40 (31-59)	0.000
ALT, (U/L), Median (IQR)	39 (25-61)	33 (22-58)	0.012
ALP, (U/L), Median (IQR)	93.5 (66.25-125.5)	79 (64-99)	0.093
GGT, (U/L), Median (IQR)	50.5 (25.25-80)	34 (21-67)	0.002
Albumin, (g/dL), Median (IQR)	3.5 (3.1-3.7)	4 (3.6-4.4)	0.000
CRP, (mg/L), Median (IQR)	18.15 (5.23-71.12)	15.9 (5-57.4)	0.249
D-Dimer, (µg/mL), Median (IQR)	0.62 (0.43-1.19)	0.69 (0.44-1.21)	0.403
ANC x 10^9^/L, Median (IQR)	5.05 (3.6-7,37)	4.4 (3.1-6.4)	0.469
ALC x 10^9^/L, Median (IQR)	1.6 (1-2.6)	1.6 (1.0-2.6)	0.077
AEC x 10^9^/L, Median (IQR)	0.1 (0.07-0.2)	0.1 (0.075-0.28)	0.017
Ferritin (µg/L), Median (IQR)	220.5 (68.9-422.5)	159 (63-341.5)	0.079
Fatty liver; n (%)	55 (22.6%)	759 (20.3%)	0.381

Two hundred forty-three patients died from the illness while 3740 patients survived the illness. Older age, male sex, elevated AST, ALT, and GGT were associated with higher mortality. Similarly, patients who died during hospital stay had lower serum albumin levels and AEC. However, multivariate regression analysis showed that only older age, male sex, and low serum albumin levels were associated with higher mortality (Table 3).

**Table 3:** Multivariate analysis of factors related to mortality in COVID-19 infected patients GGT: gamma-glutamyl transpeptidase; AST: aspartate transaminase; ALT: alanine transaminase; ESR: erythrocyte sedimentation rate; INR: international normalized ratio; CRP: C-reactive protein; WBC: white blood corpuscles; ALC: absolute lymphocyte count; AEC: absolute eosinophil count; NAFLD: nonalcoholic fatty liver disease; COVID-19: coronavirus disease 2019

Clinical and laboratory parameters	Unstandardized regression weight	P-value	OR	95% CI
Age	-0.053	0.000	0.948	0.934-0.968
Sex	-0.769	0.011	0.464	0.256-0.840
Serum albumin	0.938	0.000	2.556	1.825-3.578
GGT	0.000	0.772	1.000	0.997-1.002
AST	-0.001	0.477	0.999	0.997-1.001
ALT	0.001	0.589	1.001	0.996-1.007
ALC	0.000	0.196	1.000	1.000-1.000
AEC	0.001	0.205	1.001	1.000-1.002
NAFLD	0.139	0.614	1.149	0.669-1.976

Subgroup analysis was planned to determine whether the outcomes depend on baseline clinical and laboratory parameters or not. Patients were further divided into subgroups based on these factors (Tables 4, 5).

**Table 4:** Influence of NAFLD on the duration of hospital stay in COVID-19 patients depending on gender, age, and presence of diabetes mellitus NAFLD: nonalcoholic fatty liver disease; COVID-19: coronavirus disease 2019

Demographic and clinical parameters	Duration of hospital stay (in days) in NAFLD (N=814)	Duration of hospital stay (in days) in non-NAFLD (N=3169)	P-value
Male	10.7±7.3	10.7±7.2	0.821
Female	10.51±6.3	10.4±4.5	0.879
Elderly (Age ≥ 60 years)	10.9±5.6	10.7±5.2	0.822
Non-elderly (<60 years)	10.5± 7.5	10.6±7.0	0.873
Presence of diabetes mellitus (n,%)	10.9±4.6	10.7±5.9	0.8

**Table 5:** Influence of NAFLD on mortality in COVID-19 patients depending on gender, age, and presence of diabetes mellitus NAFLD: nonalcoholic fatty liver disease

Demographic and clinical parameters	Mortality in NAFLD,N=55, (n,%)	Mortality in non-NAFLD N=188 (n,%)	P-value
Male (n, %)	43 (17.6%)	158 (65%)	0.684
Female (n, %)	12 (4.9%)	30 (12.3%)	0.006
Elderly (age ≥ 60 years) (n, %)	25 (10.2%)	106 (43.6%)	0.771
Non-elderly (<60 years) (n,%)	30 (12.3%)	82 (33.7%)	0.096
Presence of diabetes mellitus (n,%)	53 (96.3%)	180 (95.7%)	1.00

Among the patients who died during hospitalization, 43 (17.6%) males and 12 (4.9%), females had NAFLD. In contrast, 158 (65%) males and 30 (12.3%) females had no fatty liver. Females without fatty liver were found to have a significantly higher mortality risk than females with fatty liver (p=0.006). Surprisingly, these patients were younger and had lower GGT levels.

Next, the mean duration of hospital stay was compared between patients with NAFLD and non-NAFLD with respect to age, sex, and presence of diabetes mellitus. However, no significant difference in the mean duration of hospital stay was noted with respect to age, sex, or presence of DM. Deranged liver functions were also analyzed as the predictor of mortality (Table 6) and mean duration of hospital stay (Table 7).

**Table 6:** Influence of NAFLD on mortality in COVID-19 patients based on transaminases and GGT levels NAFLD: non-alcoholic fatty liver disease; ALT: alanine transaminase; AST: aspartate transaminase; GGT: gamma-glutamyl transpeptidase; ULN: upper limit of normal; COVID-19: coronavirus disease 2019

Elevated liver enzymes and their effect on mortality	Mortality with NAFLD (n,%)	Mortality without NAFLD (n,%)	P-value
ALT> 2 ULN (n,%)	5 (5%)	12 (12.1%)	0.943
ALT< 2 ULN(n,%)	13 (13.1%)	69 (69.6%)	0.460
P-value	0.390	0.547	-------
AST > 2 ULN(n,%)	7 (7.0%)	18 (18.1%)	0.820
AST < 2 ULN(n,%)	11 (11.1%)	63 (63.6%)	0.306
P-value	0.016	0.010	--------
GGT >2ULN(n,%)	7 (7%)	18 (18%)	0.588
GGT <2ULN(n,%)	11 (11%)	64 (64%)	0.557
P-value	0.545	0.199	-------

**Table 7:** Influence of NAFLD on the duration of hospital stay in patients with COVID-19 based on transaminases and GGT levels NAFLD: non-alcoholic fatty liver disease; ALT: alanine transaminase; AST: aspartate transaminase; GGT: gamma-glutamyl transpeptidase; ULN: upper limit of normal; COVID-19: coronavirus disease 2019

Elevated liver enzymes and their effect on the duration of hospital stay	Duration of hospital stay (days) with NAFLD	Duration of hospital stay (days) without NAFLD	P-value
ALT> 2 ULN	10.0 ±4.4	10.3±5.4	0.580
ALT< 2 ULN	10.7±8.2	10.6±7.4	0.740
P-value	0.360	0.533	------------
AST > 2 ULN	10.3±4.7	11.1±6.2	0.218
AST< 2 ULN	10.6±8.1	10.4± 7.4	0.613
P-value	0.682	0.126	------------
GGT >2ULN	10.4±4.8	11.1± 6.2	0.156
GGT <2ULN	10.7±8.7	10.4± 7.4	0.535
P-value	0.639	0.093	-------------

Patients who died during hospitalization were first divided on the basis of the presence or absence of NAFLD. Afterward, patients in both groups were divided into subgroups based on levels of AST, ALT, and GGT. No statistically significant difference in mortality was noted between NAFLD and non-NAFLD groups with respect to deranged liver functions. Similarly, no difference in the mean duration of hospital stay was noted between both groups.

However, significantly lower mortality was noted in patients with NAFLD who had significantly elevated AST levels (two times upper limit of normal (ULN) compared to patients with AST levels less than 2ULN (7% vs 11%; p=0.016)). A similar observation was also noted in patients without NAFLD. Patients without NAFLD who had AST levels less than 2ULN had higher mortality compared to the patients with AST levels more than 2ULN (18.1% vs 63.6%; p=0.010) ( Table 6). Multivariate analysis also confirmed this finding (Table 8).

**Table 8:** Multivariate analysis of factors related to mortality in COVID-19 patients with low AST levels (<2ULN) irrespective of the presence of fatty liver GGT: gamma-glutamyl transpeptidase; AST: aspartate transaminase; ALT: alanine transaminase; ULN: upper limit of normal; COVID-19: coronavirus disease 2019

Clinical and laboratory parameters	Unstandardized regression weight	P-value	OR	95% CI
Age	-0.052	0.000	0.949	0.932-0.967
Sex	-0.684	0.041	0.504	0.262-0.973
Serum albumin	0.732	0.000	2.080	1.377-3.140
GGT	-0.003	0.189	0.997	0.994-1.001
AST	-0.031	0.001	0.969	0.997-1.001
ALT	0.010	0.098	1.010	0.998-1.022

## Discussion

Our study analyzed the outcomes of COVID-19 infection in patients with or without NAFLD. We found out that patients with COVID-19 and NAFLD have neither increase in mortality nor an increased duration of hospital stay. It was also noted that serum AST, ALT, and GGT levels were significantly higher in patients with NAFLD. However, none of these parameters were found to be associated with mortality or a longer duration of hospital stay. Multivariate analysis revealed only older age, male sex, and low serum albumin levels were associated with higher overall mortality. Interestingly, subgroup analysis showed that females without NAFLD were at a high risk of mortality (p=0.006). Patients with lower baseline AST levels (<2ULN) had higher mortality compared to patients with significantly elevated AST (>2 ULN) levels irrespective of the presence of fatty liver. These findings are in stark contrast with two of the previous studies, which included patients with COVID-19 and NAFLD and found a positive correlation between the presence of fatty liver and severe illness [13-15]. However, in these studies defining fatty liver was based on the consensus definition of metabolism-associated fatty liver disease (MAFLD), which needs to be validated further and may not hold good in COVID-19 infected individuals. It is also well known that COVID-19 illness is often associated with elevated transaminases as a part of systemic illness, virus-mediated hepatic injury, or the use of hepatotoxic drugs [16]. Thus using AST and ALT to define fatty liver may actually be fallacious in these patients, which were done in some of the studies that found a positive correlation between fatty liver and severe infection in COVID-19 infected patients [17,18]. In agreement with our study, one previous study by Wang et al. has shown no relationship between the severity of COVID-19 and NAFLD, although they have shown higher mortality in patients with NAFLD having normal body mass index [19]. Similarly, the study by Madan et al. also concluded that fatty liver may not be associated with high morbidity and mortality in patients with COVID-19 pneumonia [10]. In the latter two studies, fatty liver was diagnosed by ultrasonography and NCCT features, which were probably a more objective way of determining fatty liver [10,19]. However, in contrast to all these studies, ours is a large single-center study that included COVID-19-infected hospitalized patients. Defining NAFLD in our study was also objective and straightforward as liver attenuation was used to define fatty liver, which is not affected by the current disease state of infected individuals. Importantly, older age and male sex were associated with higher mortality independent of the presence of fatty liver as demonstrated in most of the studies [20]. 

The retrospective study by Wang et al. analyzed the association of NAFLD with susceptibility and outcomes of COVID-19 infected patients. They found out that 39.4% of patients with COVID-19 had NAFLD, which was higher than the prevalence in the Chinese population [19]. This points toward the higher susceptibility of NAFLD patients to COVID-19 infection. In contrast, in our study, the prevalence of NAFLD in COVID-19-infected individuals was similar to other Indian studies [7]. It can be explained by the fact that ultrasonography (USG) was used in the previous study, which has more sensitivity and specificity relative to NCCT to diagnose fatty liver. Although the use of NCCT to detect fatty liver is pretty straightforward and objective, both USG and NCCT might miss the patients with mild fatty liver [21]. In another similar study by Mahamid et al., the prevalence of fatty liver by NCCT scan was about 31% [9]. In both these previous studies, patients with NAFLD had a higher incidence of DM, obesity, and hypertension. However, in our study prevalence of DM was similar in both groups. The prevalence of obesity and hypertension could not be determined due to a lack of documentation in the clinical records. 

Similar to previous studies, we found significantly elevated AST and ALT levels in patients with NAFLD [22,23]. In addition to this, serum GGT levels were also significantly elevated. It may suggest that patients with NAFLD may have a higher incidence of liver injury as compared to non-NAFLD patients. Elevated liver enzymes have been documented in 15-58% of patients with COVID-19 infection [24]. Some of the studies including patients without NAFLD have positively correlated elevated transaminases with the severity of COVID-19 illness [25,26]. However, similar to ours, the study by Wang et al. (included patients with NAFLD) and Madan et al. didn’t find a significant correlation between elevated transaminases and severe events or mortality [10,19]. Surprisingly in sub-group analysis, we found a significantly higher number of patients who died during hospital stay (In both NAFLD and non-NAFLD groups) actually had lower AST levels at admission. In contrast to ALT, AST can be elevated in multiple non-hepatic conditions including hemolysis and myopathy. Thus, levels of AST may correlate with systemic inflammation of COVID-19 illness rather than liver injury per se. Moreover, elevated transaminases in COVID illness are multifactorial (related to the use of various hepatotoxic drugs, underlying liver disease, ethanol abuse, or underlying inflammatory process per see, and thrombotic microangiopathy) and may not always reflect severe illness [26]. Though we didn’t look for severity of illness, elevated transaminases in our study were not associated with increased mortality or increased duration of hospital stay. In addition to elevated transaminases, patients with NAFLD had significant elevation of serum GGT levels similar to a previous study in patients without NAFLD [27,28]. The presence of angiotensin-converting enzyme-2 (ACE-2) receptors in cholangiocytes may be responsible for elevated GGT levels [27,28]. Moreover, though previous studies have shown an association between elevated GGT and severe pneumonia/prolonged hospital stay [28], similar results were not replicated in our study. It can be explained by the fact that these studies were conducted without considering the presence or absence of fatty liver. Patients with NAFLD per se may also have elevated GGT irrespective of a COVID-19 positive state, which is a risk factor for developing advanced fibrosis.

In the study by Mahamid et al., patients with NAFLD had higher lymphopenia at admission, and the presence of lymphopenia correlated with the severity of the illness [9]. The presence of lymphopenia at admission has also been shown to correlate with the severity of COVID-19 pneumonia in a previous meta-analysis [29]. However, in our study, lymphocyte count was similar in both the groups and lymphocyte count was not associated with increased mortality or hospital stay in multivariate analysis. Absolute lymphocyte counts and AEC were compared between patients with and without mortality irrespective of the presence or absence of fatty liver. Low AEC was associated with higher mortality, which was also similar to previous studies [30]. However, multivariate regression analysis failed to show higher mortality in patients with low AEC levels.

Another interesting finding in our study was that females without NAFLD were at a higher risk of mortality compared to those with NAFLD. At present, we are not able to explain this finding. However, data regarding the severity of illness, ICU stay, ventilator requirement, and presence of other comorbidities were not available to us, which might have contributed to these results. Future prospective studies are needed to confirm this finding.

The main limitation of this study is that data collection was dependent on the medical officers looking after the patients as gastroenterologists/hepatologists were not managing the patients on daily basis. Some of the laboratory data were not recorded due to the pressure of workload when admission rates were high. This could have caused a few inaccurate data entries. However, the large number of patients included in the study would have neutralized some of these limitations. 

## Conclusions

To conclude, the presence of NAFLD per se may not be associated with higher mortality or longer hospital stay in COVID-19-infected patients. The etiology of elevated liver enzymes in COVID-19-infected patients is multifactorial and may not be associated with severe disease in COVID-19-infected patients with fatty liver as these patients might have elevated baseline liver enzyme levels. Surprisingly, females without NAFLD may be at higher risk of mortality, although future prospective studies are needed to validate this finding further. Moreover, older age and male sex may be the two most important predictors of mortality.
